# Curcumin-primed human BMSC-derived extracellular vesicles reverse IL-1β-induced catabolic responses of OA chondrocytes by upregulating miR-126-3p

**DOI:** 10.1186/s13287-021-02317-6

**Published:** 2021-04-29

**Authors:** Shushan Li, Sabine Stöckl, Christoph Lukas, Marietta Herrmann, Christoph Brochhausen, Matthias A. König, Brian Johnstone, Susanne Grässel

**Affiliations:** 1 Department of Orthopaedic Surgery, Experimental Orthopaedics, Centre for Medical Biotechnology (ZMB/Biopark 1), University of Regensburg, Regensburg, Germany; 2 Department of Orthopaedic Surgery, The First Affiliated Hospital of Zhengzhou University, Zhengzhou, China; 3 IZKF Group Tissue Reg. in Musculoskeletal Dis., University Hospital & Bernhard-Heine-Centrum for Locomotion Res, University of Würzburg, Würzburg, Germany; 4 Institute of Pathology, University of Regensburg, Regensburg, Germany; 5 Department of Orthopaedic Surgery, Asklepiosklinikum, Bad Abbach, Germany; 6 Department of Orthopaedics and Rehabilitation, Oregon Health & Science University, Portland, OR USA

**Keywords:** BMSC, Curcumin, Extracellular vesicles, IL-1β, Osteoarthritis, Pro-inflammatory signaling pathways, Chondrocytes

## Abstract

**Background:**

Curcumin has anti-inflammatory effects and qualifies as a potential candidate for the treatment of osteoarthritis (OA). However, curcumin has limited bioavailability. Extracellular vesicles (EVs) are released by multiple cell types and act as molecule carrier during intercellular communication. We assume that EVs can maintain bioavailability and stability of curcumin after encapsulation. Here, we evaluated modulatory effects of curcumin-primed human (h)BMSC-derived EVs (Cur-EVs) on IL-1β stimulated human osteoarthritic chondrocytes (OA-CH).

**Methods:**

CellTiter-Blue Viability- (CTB), Caspase 3/7-, and live/dead assays were used to determine range of cytotoxic curcumin concentrations for hBMSC and OA-CH. Cur-EVs and control EVs were harvested from cell culture supernatants of hBMSC by ultracentrifugation. Western blotting (WB), transmission electron microscopy, and nanoparticle tracking analysis were performed to characterize the EVs. The intracellular incorporation of EVs derived from PHK26 labeled and curcumin-primed or control hBMSC was tested by adding the labeled EVs to OA-CH cultures. OA-CH were pre-stimulated with IL-1β, followed by Cur-EV and control EV treatment for 24 h and subsequent analysis of viability, apoptosis, and migration (scratch assay). Relative expression of selected anabolic and catabolic genes was assessed with qRT-PCR. Furthermore, WB was performed to evaluate phosphorylation of Erk1/2, PI3K/Akt, and p38MAPK in OA-CH. The effect of hsa-miR-126-3p expression on IL-1β-induced OA-CH was determined using CTB-, Caspase 3/7-, live/dead assays, and WB.

**Results:**

Cur-EVs promoted viability and reduced apoptosis of IL-1β-stimulated OA-CH and attenuated IL-1β-induced inhibition of migration. Furthermore, Cur-EVs increased gene expression of BCL2, ACAN, SOX9, and COL2A1 and decreased gene expression of IL1B, IL6, MMP13, and COL10A1 in IL-1β-stimulated OA-CH. In addition, phosphorylation of Erk1/2, PI3K/Akt, and p38 MAPK, induced by IL-1β, is prevented by Cur-EVs. Cur-EVs increased IL-1β-reduced expression of hsa-miR-126-3p and hsa-miR-126-3p mimic reversed the effects of IL-1β.

**Conclusion:**

Cur-EVs alleviated IL-1β-induced catabolic effects on OA-CH by promoting viability and migration, reducing apoptosis and phosphorylation of Erk1/2, PI3K/Akt, and p38 MAPK thereby modulating pro-inflammatory signaling pathways. Treatment of OA-CH with Cur-EVs is followed by upregulation of expression of hsa-miR-126-3p which is involved in modulation of anabolic response of OA-CH. EVs may be considered as promising drug delivery vehicles of curcumin helping to alleviate OA.

## Introduction

Osteoarthritis (OA) is one of the most common age-related degenerative diseases of joints and is also one of the most common causes of disability in elderly individuals [1]. OA is characterized by synovial inflammation and hyperplasia, degeneration of the articular cartilage and menisci, sclerosis of the subchondral bone, and formation of osteophytes, plus different degrees of joint pain. Also affected are tendons, ligaments, and fat pads which increase in volume and number plus peri-articular muscles which develop sarcopenia over the time. These observations lead to the concept that OA affects all tissues of the joint and lead to a dysfunction of the whole joint [2]. However, OA pathogenesis is not fully understood, and no regenerative treatment has been approved to prevent or slow down the disease progression. Total joint replacement is still considered to be the most effective treatment for end-stage OA [3], but the risks of periprosthetic joint infections, aseptic loosening, revision surgery, and other complications have become more and more evident, especially in elderly patients [4, 5]. Thus, it is desirable to find novel therapeutic approaches to protect articular cartilage and other joint tissues and slow down OA progression at the early stage of the disease.

**Fig. 1:**
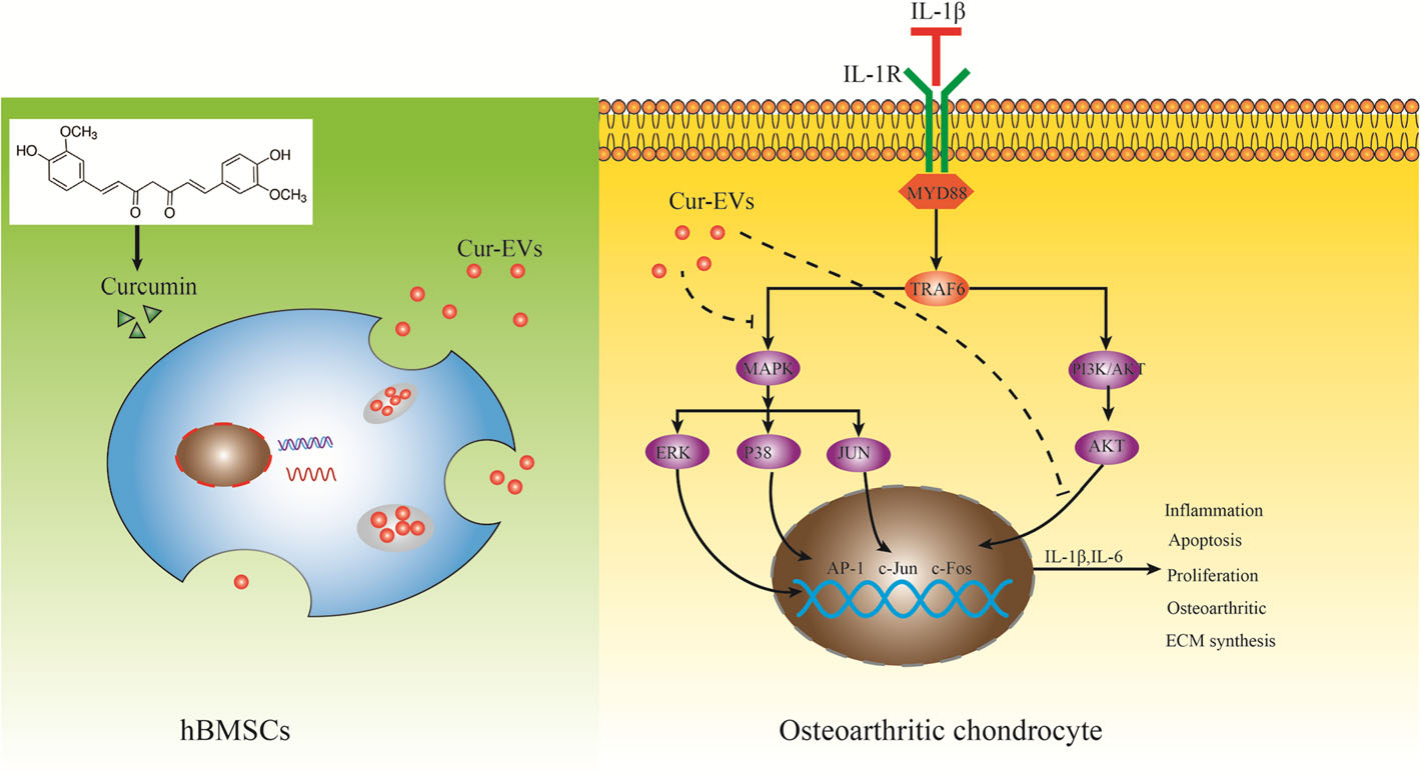
Experimental outline of IL-1β-induced osteoarthritic chondrocytes (OA-CH) treated with curcumin-primed hBMSC-derived EVs (Cur-EVs). The aim of this study was to evaluate the effect of Cur-EVs on OA-CH. OA-CH were stimulated with IL-1β to establish a standard in vitro OA model. Cur-EVs were used as a cell-free therapeutic approach to treat IL-1β-stimulated OA-CH. We hypothesize that Cur-EVs could reverse the catabolic effects induced by IL-1β in OA-CH by inhibiting inflammatory related signaling pathways

Curcumin is a kind of a natural polyphenol compound derived from turmeric [6] (Fig. 1). It is reported that curcumin has anti-osteoarthritic and anti-inflammatory effects and qualifies thus a potential candidate for the treatment of OA [7, 8]. However, due to its slow intestinal-liver metabolism, low stability, quick systemic elimination, and its hydrophobic property resulting in low solubility, curcumin has limited bioavailability [9–11].

Extracellular vesicles (EVs) are nanoscale lipid-bound vesicles secreted into the extracellular space by almost all cell types [12], which have been characterized in pre-clinical animal and cell culture models for their therapeutic properties [13, 14]. Furthermore, since they are easily taken up by target cells through natural membrane fusion or endocytosis, EVs have been investigated as vehicles to deliver miRNA (miR) and drugs [15]. Accumulating evidence suggests that the solubility, stability, and therapeutic potential of curcumin could be enhanced by encapsulation in EVs [16–19].

miRs are noncoding, endogenous, single-stranded, small RNA molecules of 19–22 nucleotides in length, which are involved in multiple biological processes, including cell cycle control, cell metabolism, cellular differentiation, proliferation, and apoptosis via targeting the 3′-untranslated region (3’UTR) of mRNA [20–22]. Numerous pre-clinical in vitro and in vivo studies using OA models have indicated that specific miR profiles are associated with OA [23–25]. For example, miR-146, one of the earliest discovered miRs in the articular cartilage, appears to be involved in modulating inflammatory pathways in OA pathology [26]. The growing number of miRs is now reported to play critical roles in modulating molecular mechanisms involved in cartilage matrix degradation [27–30].

IL-1β is well known as one of the most catabolic members of the pro-inflammatory cytokine family, regulating inflammatory and immune responses in OA pathology [31]. In the present study, we used IL-1β stimulation of chondrocytes obtained from OA-patients (OA-CH) as a standard in vitro OA model to induce catabolic effects resembling metabolic changes in OA pathology. Subsequently, we determined modulating effects of curcumin-primed human bone marrow-derived mesenchymal stromal cell (hBMSC)-derived EVs (Cur-EVs) on IL-1β-stimulated OA-CH. To investigate the molecular mechanism of putative anti-catabolic functions of Cur-EVs, we analyzed miR microarray profiles from public datasets and identified a role of hsa-miR-126-3p in metabolism of IL-1β-induced OA-CH. We provide evidence that the protective action of Cur-EVs regarding catabolic responses of IL-1β-induced OA-CH is potentially mediated via upregulating the expression of hsa-miR-126-3p, which is capable of inhibiting activation of Erk1/2, PI3K/AKT and p38 MAPK signaling.

## Materials and methods

Experimental outline and hypothesis of this study are shown in Fig. 1.

### Ethical statement

The use of human material has been approved by the local ethics committee (No. 14-101-0189; Ethikkommission, University of Regensburg, email: ethikkommission@klinik.ukr.de), and the written consent of all patients has been obtained.

### Preparation of curcumin solutions

Curcumin (#C7727-500MG; Sigma-Aldrich, Germany) stock solution (100 mM) was prepared using DMSO (#A994.2; ROTH, Karlsruhe, Germany) as a solvent. Curcumin stock solution was diluted in cell culture medium (hBMSC were cultured in StemMACS Expansion Medium supplemented with 0.2% MycoZap and OA-CH were cultured in DMEM F12 medium, supplemented with 10% FCS and 1% penicillin–streptomycin) to achieve different final concentrations. The pH of the curcumin solution was adjusted to 7.35–7.45 before application. DMSO alone was used as the vehicle control.

### Isolation and culture of hBMSC and human chondrocytes

Human bone marrow-derived mesenchymal stromal cells (hBMSC) were obtained from nine patients (mean age 62.8 ± 7.9 years, range 50–72 years, female 55.6%) who underwent total hip arthroplasty surgery due to OA. hBMSC were harvested by density gradient centrifugation according to established protocols [32, 33]. Subsequently, hBMSC were expanded for three passages in StemMACS Expansion Medium (Miltenyi Biotec, Bergisch Gladbach, Germany) supplemented with 0.2% MycoZap (#923c1069, Lonza, Switzerland) before use. Human articular cartilage biopsies were obtained from fifteen OA patients (mean age 66.4 ± 7.2 years, range 56–79 years, female 73.2%) who underwent total knee arthroplasty surgery. Healthy (non-OA) articular cartilage was obtained from the knee joints of five cadavers (mean age 24 ± 6.3 years, range 17–34 years, female 20.1%). Cartilage biopsies were cut into small pieces after being dissected from subchondral bone and digested with 0.2% type II collagenase in Dulbecco’s modified Eagle’s medium (DMEM, #D8437, Sigma, UK). After 18 h digestion, chondrocytes were pelleted, seeded in T175 flasks, and expanded in DMEM F12 medium, supplemented with 10% FCS and 1% penicillin–streptomycin until 70–80% confluency.

### EV depletion of FCS

For the generation of EV-depleted FCS, FCS (fetal calf serum) (#F7524, Sigma-Aldrich GmbH; Germany) was diluted in α-MEM (#M8042, Sigma-Aldrich GmbH; Germany) medium to a final 20% concentration and subjected to sequential ultracentrifugation steps at 120,000×*g* (L-90 K, Beckman Coulter, Brea; USA) at 4 °C for 18 h [34]. EVs depleted FCS (FCS^depl-uc^) was stored at − 20 °C in α-MEM for use in cell culture experiments.

### Cur-EV isolation

2 × 10^6^ hBMSC (passage 3) were seeded in triple T175 flasks (#132867; ThermoFisher, Germany) with StemMACS Expansion Medium (Miltenyi Biotec, Bergisch Gladbach, Germany) supplemented with 0.2% MycoZap (#923c1069, Lonza, Switzerland). Expansion medium was exchanged with α-MEM (M8042; Lot: RNBH2983. Sigma-Aldrich GmbH, Steinheim; Germany) medium, supplemented with 10% FCS^depl-uc^, 10 μM curcumin, and 1% penicillin–streptomycin when cells were 80% confluent. After 48 h incubation, differential centrifugation and ultracentrifugation steps were applied to pellet Cur-EVs from curcumin-primed hBMSC conditioned culture supernatant according to protocols previously published [34, 35]. Briefly, we applied sequential centrifugation steps for 10 min at 300×*g* to remove cells, followed by 10 min at 2000×*g* to remove dead cells and finally for 30 min at 10,000×*g* to remove cell debris. This procedure was followed by ultra-centrifugation of the supernatant at 120,000×*g* for 70 min at 4 °C and subsequently dispersing the pellet in PBS, after which the solution was filtered through a 0.22-μm filter. Finally, the Cur-EVs were pelleted at 120,000×*g* for 70 min at 4 °C and the pellet was resuspended in the presence of 25 mM Trehalose (#8897.1, ROTH, Germany) in PBS and stored at − 80 °C for use in the experiments. The concentration of Cur-EVs was determined by a BCA Protein assay according to manufacturer’s protocol (#23227, Thermo Scientific, Rockford; USA). α-MEM medium, supplemented with 10% FCS^depl-uc^ and 10 μM curcumin, underwent the same isolation procedure by ultracentrifugation as Cur-EVs and is defined as Cur-medium^ultra^. It functions as a control for the Cur-EV effects.

### hBMSC labeling and Cur-EV uptake test using PKH26

1 × 10^7^ hBMSC (passage 3) were labeled with PKH26 (Sigma-Aldrich, St. Louis; USA) according to the manufacturer’s protocol. The PKH26-labeled hBMSC were cultured in α-MEM medium, supplemented with 10% FCS^depl-uc^, 10 μM curcumin, and 1% penicillin–streptomycin. PKH26-labeled hBMSC-derived EVs were collected and purified as described in the “Cur-EV isolation” section.

1 × 10^4^ OA-CH (passages 2–4) were seeded in 8-well CultureSlides (#354118, Falcon, USA) with DMEM F12 medium (supplemented with 10% FCS, 1% penicillin–streptomycin). After 12 h, cells were incubated with purified PKH26-labeled Cur-EVs at a concentration of 10 μg/ml. After 12 h incubation, OA-CH were stained with Phalloidin (#ab235137, Abcam, UK) and DAPI (#D3571, ThermoFisher, Germany). Internalization of PKH26-labeled hBMSC-EVs was visualized and documented by fluorescence microscopy (#BX61, OLYMPUS, Japan).

### CellTiter-Blue Cell Viability Assay

The CellTiter-Blue Cell Viability Assay (#G8080, Promega, Germany), assessing the metabolic capacity of cells, was performed to determine cell viability according to the manufacturer’s protocol. 5 × 10^3^ hBMSC (passage 3, cultured in StemMACS Expansion Medium supplemented with 0.2% MycoZap) or 5 × 10^3^ OA-CH (passages 2–4, cultured in DMEM F12 medium, supplemented with 10% FCS and 1% penicillin–streptomycin) were seeded into 96-well plates. After 24 h, medium was exchanged for medium containing appropriate treatment agents (curcumin, IL-1β, EVs, Cur-EVs, Cur-medium^ultra^, miR-126-3p mimic and miR-126-3p inhibitor). After an additional 24 h, the amount of resazurin reduced to resorufin (pink and highly fluorescent) indicating viable cells was determined at 579/584 nm with a Tecan ELISA reader (Maennedorf, Switzerland).

### Caspase-3/7 assay

Caspase-3/7 enzymatic activity, which correlates with cell apoptosis, was quantified using an Apo-ONE Homogeneous Caspase-3/7 assay kit (#G7791, Promega Corporation, Madison; USA) according to the manufacturer’s instructions. After 24 h incubation, a non-fluorescent caspase substrate (Z-DEVDR110), added to 1 × 10^4^ hBMSC (passage 3, cultured in α-MEM expansion medium, supplemented with 1% penicillin–streptomycin) or 1 × 10^4^ OA-CH (passages 2–4,^,^cultured in DMEM F12 medium, supplemented with 1% penicillin–streptomycin), was cleaved into fluorescent molecules with an emission maximum at 521 nm and evaluated with a Tecan ELISA reader (Maennedorf, Switzerland).

### Live/dead cell staining assay

Calcein-AM (#17783-1MG, sigma; USA) and ethidium homodimer (46043-1MG-F, Sigma; USA) were utilized for simultaneous fluorescence detection. 2 × 10^4^ hBMSC (passage 3) or 2 × 10^4^ OA-CH (passages 2–4) were seeded in 24 well plates for 24 h, before Calcein-AM (3 μM) and ethidium homodimer (2 μM) were added to the culture medium. hBMSC were cultured in StemMACS Expansion Medium (supplemented with 0.2% MycoZap), and OA-CH were cultured in DMEM F12 medium, supplemented with 10% FCS and 1% penicillin-streptomycin). The living (green) and dead (red) cells were determined using a fluorescence microscope (#Eclipse TE2000-U, Nikon, Japan) and Image J software (National Institutes of Health, USA) for cell counting.

### Inhibition of Erk1/2, p38 MAPK, and PI3K/AKT signaling in OA-CH

2 × 10^5^ OA-CH (passages 2–4) were cultured in 6-well plates with DMEM F12 expansion medium (supplemented with 10% FCS, 1% penicillin–streptomycin), until reaching 60% confluency, then expansion medium was exchanged for DMEM F12 medium (supplemented with 1% penicillin–streptomycin). After 24 h, OA-CH were treated with 10 μM U0126 (Erk inhibitor, #9903, Cell signaling, Germany) in DMSO, 10 μM LY294002 (PI3K inhibitor, #9901, Cell signaling, Germany) in DMSO or 10 μM SB202190 (p38 MAPK inhibitor, #8158, Cell signaling, Germany) in DMSO for 30 min, followed by stimulation with IL-1β (1 ng/ml) for 30 min, prior to protein isolation (see 2.15). Additionally, 5 × 10^3^ OA-CH (passages 2–4) were cultured in 96-well plates with expansion DMEM F12 medium (supplemented with 10% FCS and 1% penicillin–streptomycin) for 24 h. Afterwards, expansion medium was exchanged with DMEM F12 medium (supplemented with 1% penicillin–streptomycin) and after another 24 h, OA-CH were treated with 10 μM U0126, LY294002 or SB202190 for 30 min, followed by stimulation with IL-1β (1 ng/ml) for 24 h. Cells were then subjected to CellTiter-Blue Cell Viability Assay, Caspase-3/7 assay, and live/dead assay.

### IL-1β stimulation of OA-CH and EV/Cur-EV treatment

2 × 10^5^ OA-CH (passages 2–4) were cultured in 6 well plates with DMEM F12 medium (supplemented with 10% FCS and 1% penicillin–streptomycin), stimulated with IL-1β (1 ng/ml) (MAN0004230, ThermoFisher; USA) for 24 h, and incubated with control EVs or Cur-EVs (both 10 μg/ml) for 24 h before being prepared for follow up experiments.

### RNA extraction and real-time RT-PCR analysis

Total RNA of cells was isolated using the Absolutely RNA Miniprep Kit (Agilent Technologies; USA) according to the manufacturer’s instructions and reverse-transcribed into cDNA using AffinityScript QPCR cDNA Synthesis Kit (#600559, Agilent Technologies; USA). Subsequently, qRT-PCR for assessing relative mRNA levels was performed with Brilliant III Ultra-Fast SYBR® Green QPCR Master Mix (#600882, Agilent Technologies; USA) using a MX3005P QPCR System (Agilent Technologies, Santa Clara; USA). All genes were analyzed relatively, calibrated to the expression of control groups, and normalized to GAPDH and TBP. All qPCR experiments were performed in duplicates using 10–50 ng cDNA.

For hsa-miR-126-3p expression analysis, total cellular RNA was isolated using the miRNeasy Mini Kit (#217004, QIAGEN; USA) according to the manufacturer’s instructions, and 30 ng of total RNA was reversely transcribed with the TaqMan MicroRNA Reverse Transcription Kit (#4366596, ThermoFisher; USA) using RT primers of TaqMan MicroRNA Assays for hsa-miR-126-3p and U6 (reference gene) (Applied Biosystems, USA). qPCR was performed in duplicates using 2 ng cDNA, TaqMan primers (hsa-miR-126-3p and U6) of TaqMan MicroRNA Assays and TaqMan Universal PCR Master Mix II, No AmpErase UNG (#4428173, Applied Biosystems). Sequences of all primers used for qRT-PCR in this study are listed in Table 1.

**Table 1:** Primer sequences for qPCR

Gene	Primer sequence
ACAN	Fwd: 5′-CTATACCCCAGTGGGCACAT-3′
	Rev:5′-GGCACTTCAGTTGCAGAAGG − 3′
BCL2	Fwd: 5′-ATGTGTGTGGAGAGCGTCAA-3′
	Rev: 5′-ACAGTTCCACAAAGGCATCC-3′
COL2A1	Fwd: 5′-CCAGATGACCTTCCTACGCC-3′
	Rev: 5′-TTCAGGGCAGTGTACGTGAAC-3′
SOX9	Fwd:5′-GTACCCGCACTTGCACAAC-3′
	Rev: 5′-TCTCGCTCTCGTTCAGAAGTC-3′
IL6	Fwd: 5′-CAATGAGGAGACTTGCCTGG-3′
	Rev: 5′-GCACAGCTCTGGCTTGTTCC-3′
COL10A1	Fwd:5′-CAC GTT TGG GTA GGC CTG TA-3′
	Rev: 5′-TCT GTG AGC TCC ATG ATT GC-3′
IL-1beta	Fwd: 5′-TAAGCCCACTCTACAGCTGG-3′
	Rev: 5′-GAGAGGTGCTGATGTACCAG-3′
MMP13	Fwd:5′-GACTGGTAATGGCATCAAGGGA-3′
	Rev: 5′-CACCGGCAAAAGCCACTTTA-3′
TBP	Fwd:5′-TTG TAC CGCAGCTGCAAA AT-3′
	Rev: 5′-TAT ATT CGG CGT TTC GGG CA-3′
GAPDH	Fwd: 5′-CTGACTTCAACAGCGACACC-3′
	Rev: 5′-CCCTGTTGCTGTAGCCAA AT-3′

### Nanoparticle tracking analysis

Nanoparticle tracking analysis (NTA) was performed to measure distribution of particle size and concentration in EV preparations (control-EVs and Cur-EVs) using a NanoSight NS300 (Malvern Instruments; Malvern; UK). Accuracy of NTA was confirmed with 100 nm polystyrene beads (Sigma-Aldrich, Germany) immediately before measurements. Control-EV samples were diluted 1:100 and Cur-EV samples were diluted 1:200 in PBS and measurements performed at 25 °C, five measurements of 30 s were recorded for each EV sample.

### Transmission electron microscopy

For the transmission electron microscopy (TEM), freshly isolated EVs were resuspended in cold PBS. For negative staining, 20 μl of the solution was added onto parafilm and a formvar (polyvinyl formal)-carbon coated 400 copper mesh grid (#G400-CU; Science Services, Munich, Germany) was placed on top of the fluid for 10 min at RT. The grid was incubated with 2% phosphotungstic acid (#19500; Science Services, Munich, Germany) for 1 min and dried at RT for 10 min. EVs were investigated with an acceleration voltage of 100 kV and × 100,000 magnification using a Leo 912 AB (Carl Zeiss, Oberkochen, Germany).

### Protein extraction and western blot analysis

OA-CH were washed 2 times with PBS and lysed with RIPA buffer (Thermo Scientific, Waltham, MA) containing phosphatase (#04906845001, Roche, Germany) and proteinase inhibitors (#04693116001, Roche, Germany). The concentration of cellular protein and EVs was quantified using a BCA protein kit assay (see the “Cur-EV isolation” section). Either 10 μg cell lysates or EVs was mixed with SDS-sample loading buffer, followed by boiling for 5 min at 95 °C, and subjected to a 12% SDS-PAGE. The proteins were transferred onto 0.22 μm PVDF membranes (Roche, Penzberg, Germany) after electrophoretic separation. Blot membranes were first stained with Ponceau Red solution (loading control), washed and then blocked with 5% BSA for 1 h at room temperature, and incubated with primary antibodies on a shaker overnight at 4 °C. After washing, the membranes were incubated with the appropriate horseradish peroxidase-coupled secondary antibodies (Santa Cruz Biotechnology, and Jackson Immuno Research, West Grove, PA). Protein bands were visualized using ECL detection reagents (Thermo Scientific, Germany). Image J (1.8.0, National Institutes of Health, Bethesda, USA) software was used to quantify the density of bands of phosphorylated proteins and normalize them to the total amount of protein bands. Antibodies used for this study are listed in Table 2.

**Table 2:** Antibodies for western blot

Primary antibodies	Company and catalog no.	Dilution
CD 9	ThermoFisher: 10626D	1:1000
CD 81	ThermoFisher: 10630D	1:1000
CD 63	ThermoFisher: 10628D	1:500
Phospho-P38	Cell signaling: 4511	1:1000
P38	Cell signaling: 9212	1:1000
Phospho-Erk1/2	Cell signaling: 4370	1:1000
Erk1/2	Cell signaling: 4695	1:1000
Phospho-Akt	Cell signaling: 4060	1:1000
Akt	Cell signaling: 4691	1:1000
Secondary antibody	Jackson Immuno Research: 715-036-150	1:5000

### Migration (wound healing) assay

1 × 10^4^ OA-CH (passages 2–4) were cultured in cell culture inserts (#80206, ibidi, Germany) until reaching 100% confluency. Subsequently, cell culture inserts were removed, leaving a gap to be filled by migrating cells. Cells were washed twice with PBS, and after changing to DMEM F12 medium (supplemented with 1% penicillin–streptomycin), IL-1β (1 ng/ml) and/or EVs/Cur-EVs (10 μg/ml) were added. Sequential images were captured with a camera-equipped microscope (#Eclipse TS100, Nikon, Japan) at 0 h, 24 h, 48 h, and 72 h post wounding, and closure of the wound gap was measured using Image J (1.8.0, National Institutes of Health, Bethesda, USA).

### Determination of curcumin concentration in EVs

The concentration of curcumin in Cur-EVs samples was determined using a Nanodrop 1000 spectrophotometer (Thermo Scientific, Waltham, MA) at 420 nm. Before determination, a standard curve of curcumin was established using different concentrations (0 μM, 5 μM, 10 μM, 20 μM, 50 μM, 100 μM, 200 μM) of curcumin solution. The Cur-EVs samples were lysed with RIPA buffer (Thermo Scientific,) for 5 min, sonicated twice for 10 s, and the curcumin quantity was calculated based on OD 420 nm according to the included standard curve of curcumin.

### Bioinformatics analysis

miR microarray datasets (EMTAB-5715, EMTAB-5716) were obtained from ArrayExpress (https://www.ebi.ac.uk/arrayexpress/), including 17 non-OA cartilage samples and 24 OA cartilage samples. Limma package (http://bioconductor.org/packages/release/bioc/html/limma.html) was used to normalize and screen the differentially expressed miRs. The miR expression levels with a fold change > 2 or fold change < − 2 and *p* value < 0.05 were considered to be significantly expressed. TargetScan (http://www.targetscan.org/vert_72/), miRDB (http://www.mirdb.org/), and miRTarBase (https://maayanlab.cloud/Harmonizome/resource/MiRTarBase) were used to indicate target genes of hsa-miR-126-3p. KEGG pathway enrichment analysis enriched in target genes was analyzed using the clusterProfiler (version 3.11) of R package [36].

### hsa-miR-126-3p overexpression and knockdown in OA-CH

In order to investigate the effect of hsa-miR-126-3p in IL-1β-induced OA-CH, hsa-miR-126-3p mimic and hsa-miR-126-3p inhibitor were transfected into the cells to overexpress or inhibit hsa-miR-126-3p. The respective RNA oligos were synthesized by Ambion, Inc. (Austin, TX, USA), and their sequences were as follows: hsa-miR-126-3p mimic: 5′-UCGUACCGUGAGUAAUAAUGCG-3′; hsa-miR-126-3p inhibitor: 5′-UCGUACCGUGAGUAAUAAUGCG-3′. OA-CH (2 × 10^5^, passages 2–4) were cultured in 6-well plates with DMEM F12 medium (supplemented with 10% FCS, 1% penicillin–streptomycin), stimulated with IL-1β (1 ng/ml) for 24 h, and then IL-1β-induced OA-CH were transfected with the hsa-miR-126-3p mimic, hsa-miR-126-3p mimic negative control (#4464058, Ambion, USA), hsa-miR-126-3p inhibitor, or hsa-miR-126-3p inhibitor negative control (#4464076, Ambion, USA) using Lipofectamine® RNAiMAX Reagent (#13778075, Invitrogen, Carlsbad, CA, USA) at a final concentration of 100 nM. Isolation of protein and RNA was performed 1 h and 24 h after transfection, respectively. Viability assay, Caspase-3/7 assay, and live/dead assay were performed 24 h after transfection.

### Statistical analysis

All data are expressed as the mean ± SD. Statistical analysis was performed using Prism 8.02 software (GraphPad Software, USA). Comparisons between two groups were analyzed by independent two-tailed Student’s *t* tests, and comparisons between more than two groups were analyzed by one-way ANOVA with Newman-Keuls multiple comparison test. Each assay was performed in replicates and repeated at least in three independent experiments. *P* < 0.05 was regarded as statistically significant.

## Results

### Cytotoxicity of curcumin regarding hBMSC and OA-CH

**Fig. 2:**
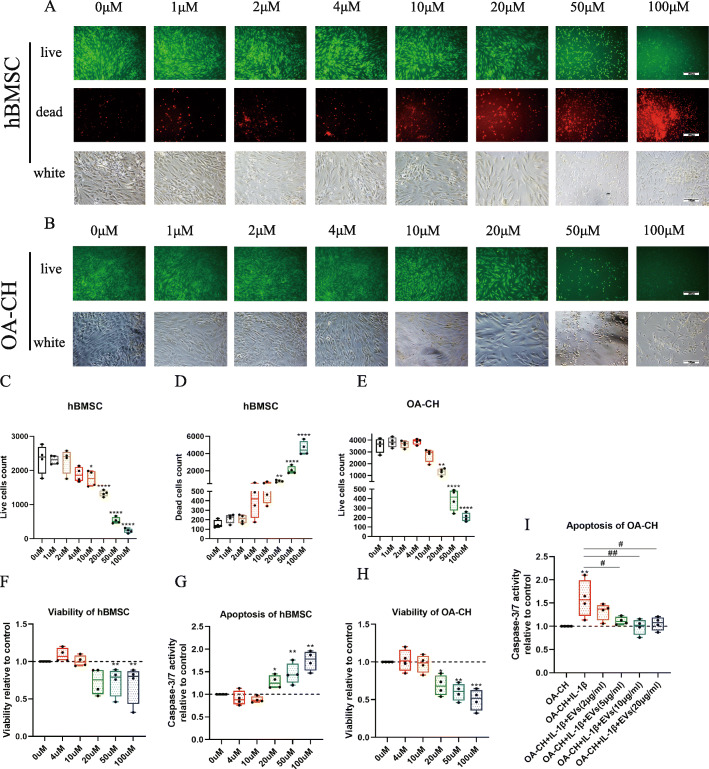
Effect of different concentrations of curcumin on cell death, viability and apoptosis of hBMSC and OA-CH. Live/dead staining, CellTiter-Blue assay, and Caspase-3/7 assay were used to determine the viability and apoptosis of hBMSC and OA-CH after treatment with different concentrations of free curcumin for 24 h. **a**, **b** Vital and dead hBMSC and OA-CH were visualized by fluorescence microscopy after labeling cells with calcein and ethidium homodimers. Living cells were labeled with calcein (green fluorescence) and dead cells were stained with ethidium homodimer (red fluorescence). Scale bar = 200 μm. **c**–**e** Quantification of live/dead staining conducted with hBMSC and OA-CH after stimulation with different concentrations of free curcumin (0–100 μM). **f**–**h** Quantification of viability and apoptosis assays conducted with hBMSC and OA-CH after treatment with different concentrations of free curcumin (0–100 μM). The 0 μM group without curcumin treatment is defined as control (DMSO only). **i** Quantification of caspase 3/7 assay conducted with IL-1β-induced OA-CH after treatment with different concentrations of EVs. The OA-CH group (no treatment) is defined as control. Difference to control: *p < 0.05; **p < 0.01; ***p < 0.001; ****p < 0.0001; ^#^difference between groups: ^#^*p* < 0,05; ^##^*p* < 0,01; 1-way ANOVA with Newman-Keuls multiple comparison test; *n* = 4

In order to test the effect of curcumin on OA-CH and find a biocompatible concentration of curcumin for incubation of hBMSC to isolate EVs containing curcumin as part of their cargo (Cur-EVs), the cytotoxicity of different curcumin concentrations was analyzed after 24 h treatment. Curcumin induced changes in the size and morphology of hBMSC and OA-CH that was particularly evident at higher concentrations (Fig. 2a, b). Concomitantly, the live cell count of hBMSC and OA-CH significant decreased and number of dead hBMSC cells increased significantly when the concentration of curcumin was 20 μM or higher (Fig. 2c–e).

Consistent with the results of the live/dead assay, treatment with curcumin at concentrations higher than 20 μM significantly reduced the viability of both hBMSC and OA-CH (Fig. 2f, h). Caspase 3/7 activity of hBMSC was strongly induced by curcumin at concentrations of 20 μM or above, correlating with increased apoptosis (Fig. 2g).

According to these data, 10 μM was considered as the most suitable concentration of curcumin for priming of hBMSC to isolate the Cur-EVs.

Furthermore, the effect of EVs on IL-1β-induced OA-CH was analyzed using a Caspase 3/7 activity assay. IL-1β-induced OA-CH were treated with different concentrations of EVs and the data revealed that EVs with a concentration of ≥ 5 μg/ml reversed increased caspase 3/7 activity of OA-CH induced by IL-1β (Fig. 2i).

### EV and Cur-EV isolation and characterization

**Fig. 3:**
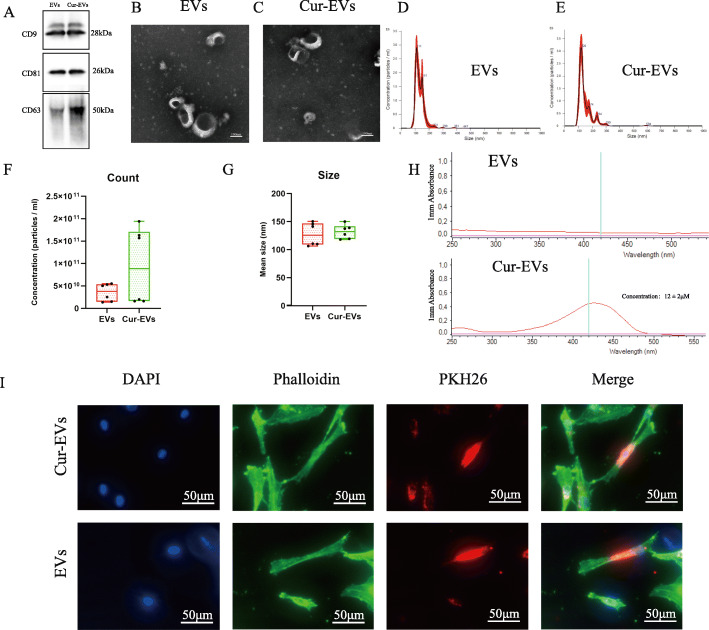
Characterization of control EVs and Cur-EVs. **a** Representative western blot image showing bands of standard surface markers (CD9, CD63, CD81) of control EV- and Cur-EV lysates. **b**, **c** Morphology of control EVs and Cur-EVs was monitored by TEM; scale bar, 100 nm. **d**, **e** Particle size distribution of control EVs and Cur-EVs was measured by NTA. **f**, **g** Quantitative comparison between control EVs and Cur-EVs in count and size measured by NTA; *n* = 3. **h** Absorbance at 420 nm of control EVs and Cur-EVs was determined with by spectrophotometry indicating presence of curcumin in Cur-EVs; *n* = 3. **i** Cell nuclei were stained with DAPI (blue) and chondrocytes were stained with Phalloidin (green) to visualize the structure of the cytoskeleton. PKH26-labeled control EVs (red) and Cur-EVs (red) were internalized by chondrocytes and visualized with fluorescent microscopy

EVs were isolated from control hBMSC and Curcumin hBMSC conditioned culture supernatants, and classical surface makers (CD9, CD63, CD81) of EVs were verified by western blotting (Fig. 3a). Morphology of control EVs/Cur-EVs was monitored by TEM, which revealed particles with an average diameter of 90–120 nm (Fig. 3b, c). Particle size distribution of control EVs/Cur-EVs was determined by NTA, which indicated the average size of both EVs samples to be approximately 120 nm, corresponding to the standard size of EVs Fig. 3d, e). Quantitative comparison of counts and size between EVs and Cur-EVs by NTA showed that there was no significant difference between these EV groups, even though number of Cur-EVs was increased by trend (Fig. 3f, g). Spectrophotometry was used to detect the presence of curcumin in Cur-EVs. Cur-EVs emitted a higher absorbance at 420 nm than control EVs, indicating the presence of curcumin in their cargo. The concentration (12 ± 2 μM, *n* = 3) of curcumin in Cur-EVs was determined according to the standard curve (Fig. 3h). In order to evaluate internalization of EVs/Cur-EVs into OA-CH, the cells were incubated with PKH26-labeled EVs/Cur-EVs for 12 h. Strong intracellular fluorescence signal (red) was detected in OA-CH cytoplasm indicating that both control EVs and Cur-EVs were taken up by OA-CH (Fig. 3i).

### Cur-EVs promoted viability and inhibited apoptosis of IL-1β-treated OA-CH

**Fig. 4:**
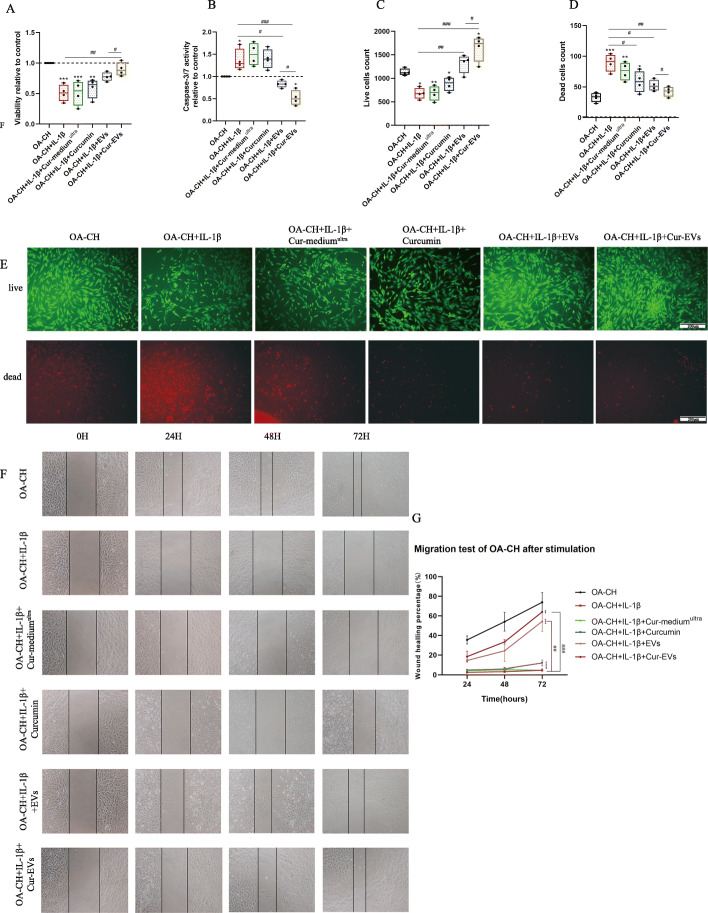
Effect of control EVs and Cur-EVs on IL-1β-induced OA-CH. **a**, **b** Cell Titer-Blue assay and Caspase-3/7 assay were used to determine viability and apoptosis of OA-CH after treatment with Cur-medium^ultra^, free curcumin (10 μM), both EV groups and IL-1β for 24 h; *n* = 4. **c**–**e** Live/dead cells were visualized by fluorescence microscopy after labeling cells with calcein and ethidium homodimers. Living cells were labeled with calcein (green fluorescence) and dead cells were stained with ethidium homodimer (red fluorescence); *n* = 4, scale bar = 200 μm. **f**, **g** A scratch (wound healing) assay was used to evaluate the effect of control EVs and Cur-EVs on migration of IL-1β-induced OA-CH. Images of gaps were taken at 0 h, 24 h, 48 h, and 72 h after treatment of cells with Cur-medium^ultra^, free curcumin (10 μM), both EV groups, and IL-1β. Wound closure rate was used to calculate the migration ability of each group; *n* = 3. All values represent mean ± standard deviation. Difference to no treatment controls (OA-CH): *p < 0.05; **p < 0.01; ***p < 0.001; ****p < 0.0001, ^#^Difference between groups: ^#^*p* < 0.05; ^##^*p* < 0.01; ^###^*p* < 0.001; 1-way ANOVA with Newman-Keuls multiple comparison test

IL-1β significantly inhibited viability and induced apoptosis of OA-CH. The viability of IL-1β-treated OA-CH was significantly higher in the presence of the control EV and Cur-EV treatment groups compared with the groups without EV treatment (Fig. 4a), whereas apoptosis of IL-1β-treated OA-CH was significantly decreased in the control EV and Cur-EV treated groups (Fig. 4b). Notably, treatment with Cur-EVs resulted in stronger reversion of the effects induced by IL-1β compared with control EVs.

Treatment with EVs and Cur-EVs increased the number of vital IL-1β-treated OA-CH significantly. Notably, the cell count of living OA-CH in the Cur-EV treated group was significantly higher than in the control EV group. Concomitantly, the number of dead OA-CH cells was significantly decreased in the IL-1β-treated group in the presence of control EVs and Cur-EVs (Fig. 4c-e). These results suggest that both control EVs and Cur-EVs could promote viability of IL-1β-treated OA-CH by reducing IL-1β-induced apoptosis, but that Cur-EVs were more effective.

Treatment of OA-CH with 10 μM free curcumin (not encapsulated in EVs) did not induce similar anabolic effects when compared to both EV groups but reduced number of dead cells (Fig. 4d). Concentration of 10 μM was chosen according to the concentration of curcumin in EVs (Fig. 3h) and to concentration titration data shown under Fig. 2b, e, h, i.

### Cur-EVs induced migration of IL-1β-treated OA-CH

A scratch (wound healing) assay was used to analyze the effect of control EVs and Cur-EVs on migration of IL-1β-treated OA-CH. Treatment with IL-1β reduced migration capacity of OA-CH significantly almost to zero (Fig. 4f). We observed a higher wound-healing (migration) rate in the OA-CH control group, EV control group, and Cur-EV group compared with the IL-1β-treated group. Additionally, the migration rate in the Cur-EV group was significantly higher than in the control EV group, whereas free curcumin only slightly induced migration rate (Fig. 4f, g).

These results indicate that EVs have a positive effect on migration capacity of OA-CH. Of note, Cur-EVs have a stronger capability to restore migration capacity of IL-1β-treated OA-CH than control EVs whereas free curcumin did not achieve comparable effects.

### Effect of Cur-EVs on gene expression of IL-1β-treated OA-CH

**Fig. 5:**
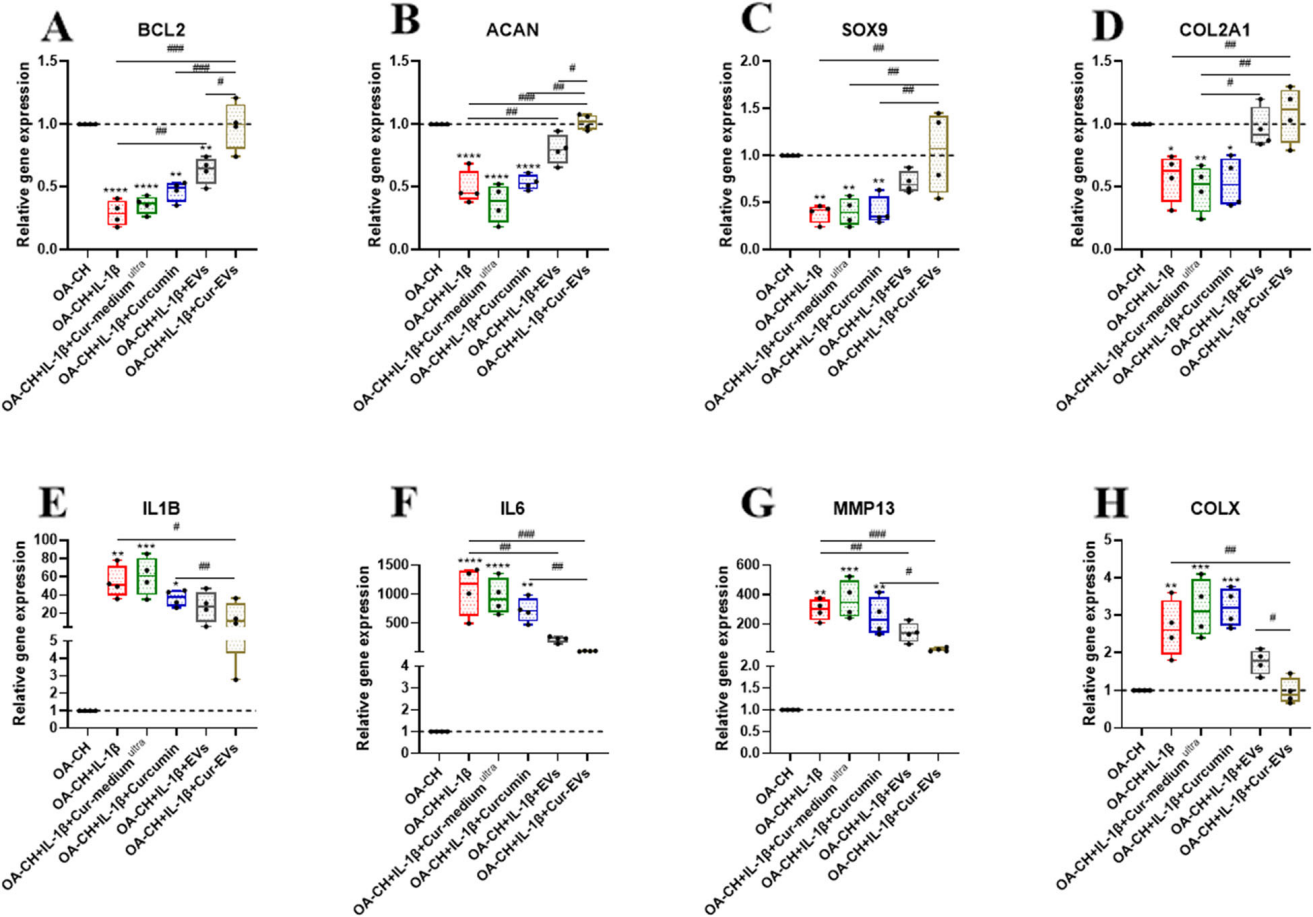
Effect of control EVs and Cur-EVs on gene expression of IL-1β-induced OA-CH. After 24 h treatment with IL-1β and/or Cur-medium^ultra^, free curcumin (10 μM), control EVs and Cur-EVs, expression levels of anabolic genes (**a**–**d** BCL2, ACAN, SOX9, COL2A1) and catabolic genes (**e**–**h** IL1B, IL6, MMP13, COL10A1) were determined with real-time RT-PCR analysis; *n* = 4. All values represent the mean ± standard deviation. Difference to no treatment controls (OA-CH): *p < 0.05; **p < 0.01;***p < 0.001; ****p < 0.0001, ^#^difference between groups: ^#^*p* < 0.05; ^##^*p* < 0.01; ^###^*p* < 0.001; ^####^*p* < 0.0001; 1-way ANOVA with Newman-Keuls multiple comparison test

qRT-PCR was performed to determine relative expression of anabolic and catabolic genes in IL-1β-treated OA-CH after treatment with control EVs and Cur-EVs. As shown in Fig. 5, expression levels of anabolic genes (BCL2, ACAN, SOX9, COL2A1) were increased in the control EVs and Cur-EVs treated groups compared with IL-1β-treated groups (Fig. 5a–d). Furthermore, Cur-EVs induced gene expression of BCL2 and ACAN significantly stronger, compared to control EVs. In contrast, expression levels of catabolic genes (IL-1beta, IL6, MMP13, COL10A1) were significantly decreased in the Cur-EVs treated groups compared to the IL-1β groups (Fig. 5e–h). Moreover, gene expression levels of IL6 and MMP13 were significantly decreased in the Cur-EV groups compared with the control EV groups. Free curcumin did not affect gene expression of anabolic genes but also reduced expression of catabolic genes except COL10A; however, it is less effective than both EV groups. These results indicated that although both control EVs and Cur-EVs induced anabolic gene expression and inhibited catabolic gene expression in OA-CH in the presence of IL-1β, the Cur-EVs had a significantly greater effect.

### hsa-miR-126-3p was downregulated in OA-CH and IL-1β-treated OA-CH compared with non-OA-CH

**Fig. 6:**
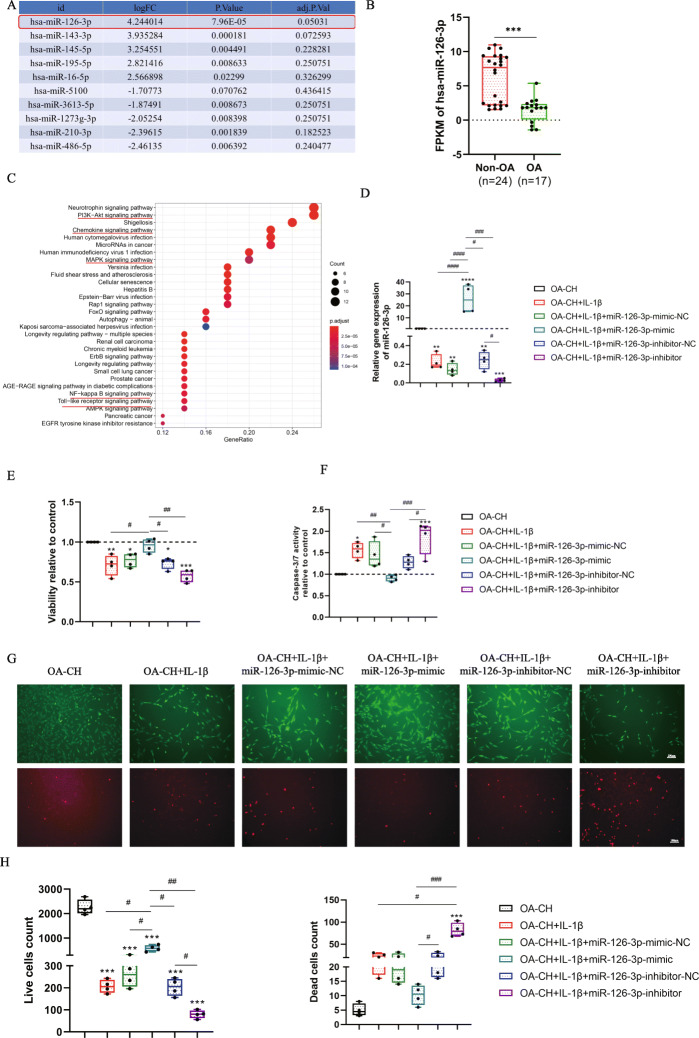
Bioinformatic analysis and gene expression of hsa-miR-126-3p in chondrocytes and the effect of hsa-miR-126-3p on the viability and apoptosis of IL-1β-induced OA-CH. **a** Top 10 differentially expressed miRs in non-OA and OA cartilage samples. LogFC:log fold change of different FPKM (fragments per kilobase of exon model per million mapped fragments) in non-OA cartilage compared with OA cartilage. **b** FPKM analysis of hsa-miR-126-3p in Non-OA and OA cartilage samples; independent two-tailed Student’s *t* tests, ****p < 0,001. **c** KEGG pathway enrichment of targeted genes of hsa-miR-126-3p. **d** Relative expression of transfected hsa-miR-126-3p mimic, inhibitor, and respective controls measured by qRT-PCR in OA-CH after IL-1β stimulation, *n* = 4. **e** Viability and apoptosis of OA-CH after treatment with IL-1β and transfection of the different miR groups; *n* = 4; **g**–**i** 24 h after treatment with IL-1β and transfection with different miR groups, live/dead OA-CH were visualized by fluorescence microscopy after labeling cells with calcein and ethidium homodimers. Living cells were labeled with calcein (green fluorescence) and dead cells were stained with ethidium homodimer (red fluorescence); *n* = 4. Difference to control (OA-CH): *p < 0.05; **p < 0.01; ***p < 0.001; ****p < 0.0001; ^#^difference between groups: ^#^*p* < 0.05; ^##^*p* < 0.01; ^###^*p* < 0.001; ^####^*p* < 0.0001; 1-way ANOVA with Newman-Keuls multiple comparison test. NC, negative control

After analysis of miRNA (miR) microarray datasets from a database, we focused on investigation of hsa-miR-126-3p as the significantly downregulated miR in OA compared to non-OA chondrocytes (Fig. 6a). We observed that expression of hsa-miR-126-3p was significant lower in chondrocytes derived from cartilage tissue of OA patients compared with chondrocytes from non-OA cartilage biopsies (Fig. 6b). Target genes of hsa-miR-126-3p were indicated by evaluation of the public database, KEGG pathway enrichment, which showed that hsa-miR-126-3p is involved in regulation of pro-inflammatory signaling pathways, including PI3K/AKT signaling pathway, chemokine signaling pathway, MAPK signaling pathway, NF-κB signaling pathway, and the Toll-like receptor signaling pathway (Fig. 6c). These data suggested that reducing expression of hsa-miR-126-3p probably plays a critical role in the pathophysiology of OA.

### hsa-miR-126-3p promoted viability and inhibited apoptosis of IL-1β-induced OA-CH

To determine whether hsa-miR-126-3p is able to regulate viability and apoptosis of IL-1β-induced OA-CH, cells were transfected with hsa-miR-126-3p-mimic-NC (NC = negative control), hsa-miR-126-3p-mimic, hsa-miR-126-3p-inhibitor-NC, and hsa-miR-126-3p-inhibitor for 24 h. As shown in Fig. 6d, the expression level of hsa-miR-126-3p was significantly upregulated by hsa-miR-126-3p-mimic and was reduced by hsa-miR-126-3p-inhibitor. Hsa-miR-126-3p-mimic also significantly promoted viability and live cell count and inhibited caspase 3/7 activity of IL-1β-treated OA-CH. Contrarily, hsa-miR-126-3p-inhibitor reduced viability and induced caspase 3/7 activity of IL-1β-treated OA-CH (Fig. 6e–i).

### Erk1/2, p38 MAPK, and PI3K/Akt signaling is induced by IL-1β in OA-CH

**Fig. 7:**
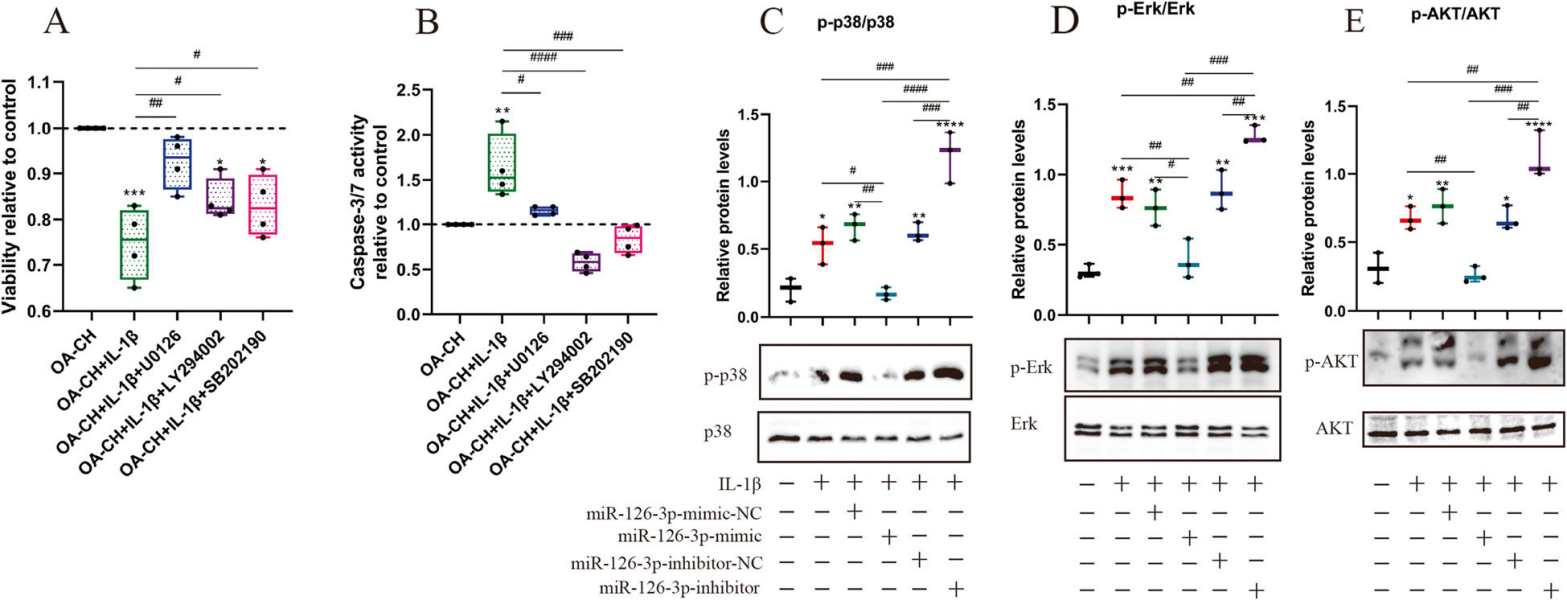
Effect of Erk1/2, p38 MAPK, and PI3K/AKT signaling on vitality of IL-1β-stimulated OA-CH and of hsa-miR-126-3p mimic on phosphorylation of Erk1/2, PI3K/Akt, and p38 MAPK in IL-1β-treated OA-CH. **a**, **b** Viability and apoptosis of OA-CH after treatment with IL-1β, U0126 (MAPK/ERK inhibitor), LY294002 (PI3K/Akt inhibitor), and SB202190 (p38 MAPK inhibitor); *n* = 4. **c**–**e** Phosphorylation levels of Erk1/2, PI3K/Akt, and p38 MAPK in OA-CH were detected by western blotting; *n* = 4. All values represent mean ± standard deviation. Difference to control: *p < 0.05; **p < 0.01; ***p < 0.001; ****p < 0.0001; ^#^difference between groups: ^#^*p* < 0,05; ^##^*p* < 0,01; ^###^*p* < 0,001; ^####^*p* < 0,0001; 1-way ANOVA with Newman-Keuls multiple comparison test

In order to investigate the involvement of Erk1/2, p38 MAPK, and PI3K/Akt signaling pathways in the catabolic responses of OA-CH, the viability and caspase 3/7 activity of IL-1β-induced OA-CH were determined after treatment with inhibitors of Erk1/2 (U0126), PI3K/Akt (LY294002), and p38MAPK (SB202190) for 24 h (Fig. 7a, b). The results suggested that U0126, LY294002, and SB202190 significantly increased viability and inhibited apoptosis of IL-1β-treated OA-CH. These data suggest that Erk1/2, PI3K/Akt, and p38 MAPK signaling pathways play critical roles in catabolic responses of OA-CH induced by IL-1β.

### hsa-miR-126-3p reduced phosphorylation of Erk1/2, PI3K/Akt, and p38MAPK in IL-1β-treated OA-CH

To determine the effect of miR-126-3p on Erk1/2, PI3K/Akt, and p38 MAPK signaling, western blotting was performed 2 h after IL-1β-induced OA-CH were transfected with hsa-miR-126-3p-mimic-NC, hsa-miR-126-3p-mimic, hsa-miR-126-3p-inhibitor-NC, and hsa-miR-126-3p-inhibitor. The phosphorylation levels of Erk1/2, PI3K/Akt, and p38 MAPK were significantly inhibited by hsa-miR-126-3p-mimic and upregulated by hsa-miR-126-3p-inhibitor (Fig. 7c–e). These results demonstrated that hsa-miR-126-3p controls the activity levels of Erk1/2, PI3K/Akt, and p38 MAPK in IL-1β-treated OA-CH.

### Cur-EVs reduced phosphorylation levels of Erk1/2, PI3K/Akt, and p38 MAPK in IL-1β-treated OA-CH via upregulating hsa-miR-126-3p expression

**Fig. 8:**
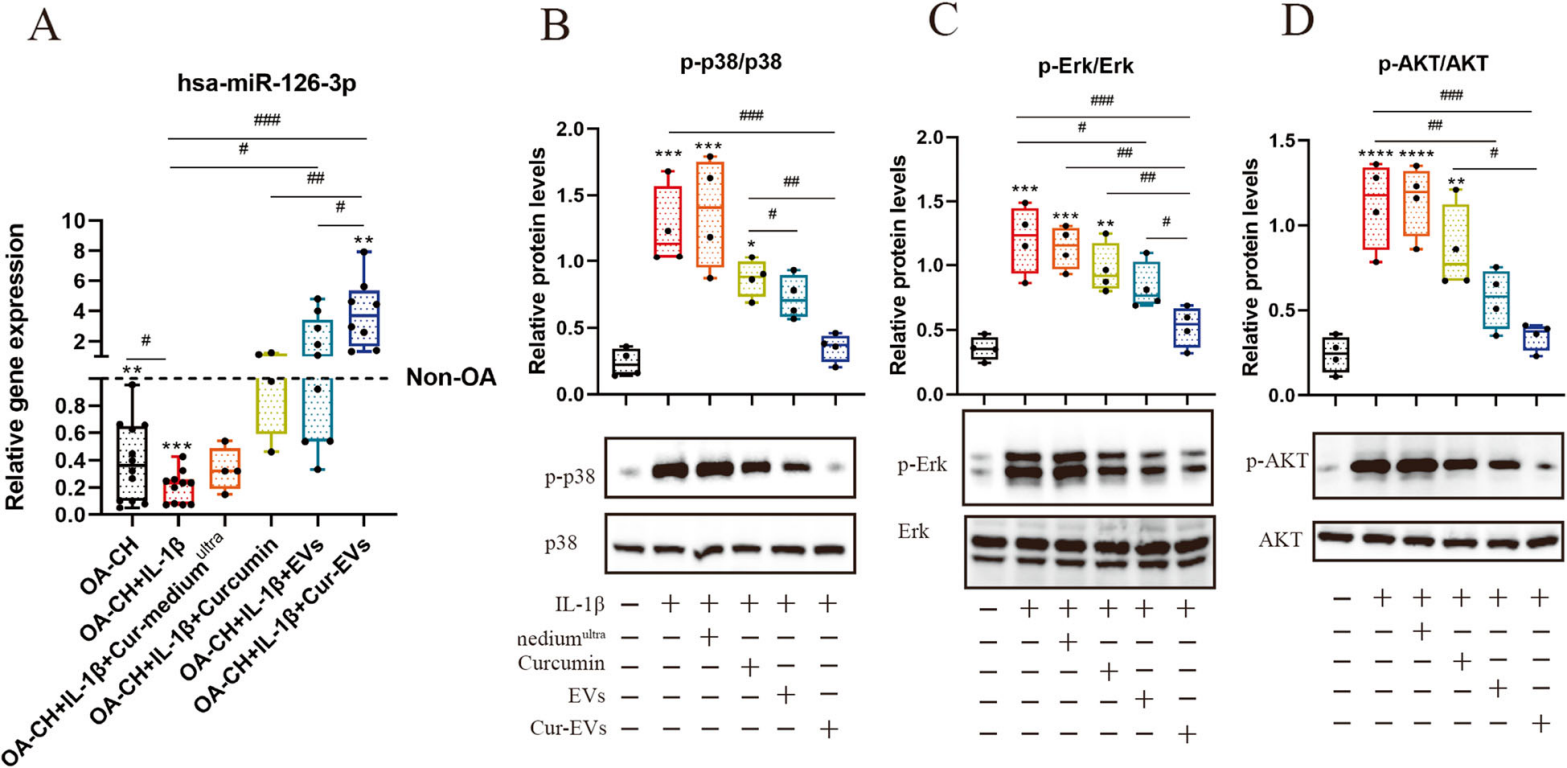
Effect of Cur-EVs on phosphorylation of Erk1/2, PI3K/Akt, and p38 MAPK in IL-1β-treated OA-CH and on gene expression of hsa-miR-126-3p. Phosphorylation levels of Erk1/2, PI3K/Akt, and p38 MAPK in OA-CH were detected by western blotting. **a** Gene expression of hsa-miR-126-3p in OA-CH, OA-CH, IL-1β-induced OA-CH in different treatment groups (Cur-medium^ultra^, free curcumin (10 μM), EVs and Cur-EVs). Difference to non-OA-CH (dotted line); **p < 0.01; ***p < 0.001, ^#^difference between groups: ^#^*p* < 0,05; ^##^*p* < 0,01; ^###^*p* < 0,001; 1-way ANOVA with Newman-Keuls multiple comparison test; *n* = 4–15. **b**–**d** Representative western blot images and quantification of phosphorylation level of Erk1/2, P13K/Akt, and p38 MAPK after densitometric analysis. All values represent mean ± standard deviation. *Compared with no treatment control (OA-CH), *p < 0.05; **p < 0.01; ***p < 0.001; ****p < 0.0001; ^#^difference between groups, ^*#*^*p* < 0,05; ^*##*^*p* < 0,01; ^*###*^*p* < 0,001; one-way ANOVA with Newman-Keuls multiple comparison test; *n* = 4

To investigate whether the hsa-miRNA-126-3p expression in IL-1β-treated OA-CH is influenced by the control EVs or Cur-EVs, qPCR was performed after 24 h of treatment with IL-1β, Cur-medium^ultra^, free Curcumin, and both groups of EVs. As shown in Fig. 8a, free curcumin, EVs, and Cur-EVs reversed the IL-1β-induced inhibition of hsa-miR-126-3p expression in OA-CH with Cur-EVs revealing the strongest effect.

To analyze the effect of Cur-EVs on components of these pro-inflammatory signaling pathways in IL-1β-treated OA-CH, western blotting was performed to evaluate the protein and phosphorylation levels of Erk1/2, PI3K/Akt, and p38 MAPK. We detected an increased phosphorylation of Erk1/2, PI3K/AKT, and p38 MAPK in IL-1β-treated OA-CH compared to controls (absence of IL-1β). In the presence of free curcumin, control EVs, or Cur-EVs, phosphorylation levels of all three kinases were reduced. Of note, in the presence of Cur-EVs, the decrease of phosphorylation of Erk1/2, PI3K/Akt, and p38 MAPK in IL-1β-treated OA-CH was stronger than after treatment with control EVs and free curcumin which shows the least effect (Fig. 8b–d). These results suggested that Cur-EVs are more effective than control EVs and free curcumin in inhibiting IL-1β-induced activation of Erk1/2, p-AKT, and p38MAPK signaling pathways in OA-CH, probably via upregulating hsa-miR-126-3p expression.

## Discussion

There is accumulating evidence that pro-inflammatory cytokines and pro-inflammatory signaling pathways are involved in the pathophysiology of OA [37]. Specifically, IL-1β, IL-6, and TNF-α have been reported to play a critical role in the progress of OA in vivo and vitro [38, 39]. Additionally, the effects of pro-inflammatory signaling by MAPK, NF-κB, and PI3K/Akt controlled pathways have been implicated in the progression of OA [40–42].

hBMSC-derived EVs were reported to exert anti-inflammatory roles in OA pathophysiology [43, 44]. Curcumin was shown to be an anti-inflammatory agent modulating MAPK, NF-κB, and PI3K/Akt signaling pathways in a rat model of OA and in cell culture experiments with OA chondrocytes [8, 45–47]. However, the bioavailability of curcumin is poor because of its low solubility, instability, and rapid systemic elimination [48]. For these reasons, curcumin was encapsulated in EVs in this study by stimulating hBMSC with curcumin and subsequently isolating the EV fraction. These curcumin-primed EVs (Cur-EVs) were used as a nanoscale delivery system of curcumin and to analyze the effects of curcumin on IL-1β-stimulated OA-CH. Our hypothesis was that we would see stronger anti-inflammatory and anti-catabolic effects of Cur-EVs compared with control EVs or free curcumin on IL-1β-stimulated OA-CH.

Curcumin effects, after being encapsulated in EVs, have been studied already in different in vivo and in vitro experimental settings [19, 49, 50]. Our characterization methods using western blotting, TEM, NTA, and internalization assays did not reveal significant differences between control EVs and Cur-EVs in terms of standard surface markers, size distribution, counts, shape, and internalization rate, which is consistent with previous reports [17, 51].

In the present study, we observed that both control EVs and Cur-EVs significantly increased viability and inhibited apoptosis of IL-1β-treated OA-CH. Our gene expression data revealed that Cur-EVs upregulated expression of anabolic genes (BCL2, ACAN, SOX9, COL2A1) and downregulated expression of catabolic genes (IL-1beta, IL6, MMP13, COL10A1) more strongly than control EVs. We assume that modulating expression of these genes is part of the underlying molecular mechanism by which Cur-EVs increased viability and reduced apoptosis of IL-1β-treated OA-CH. Of note, our results suggested that Cur-EVs exert stronger pro-viability effects, which attenuate the IL-1β-induced catabolic responses of OA-CH more effectively, in comparison with the effects of control EVs.

Previous studies demonstrated that pro-inflammatory signaling pathways play critical roles in chondrocyte catabolism and articular cartilage matrix degradation [52–54]. When we treated OA-CH with inhibitors of MAPK/Erk, PI3K/Akt13, and p38 MAPK before cells were stimulated with IL-1β, we observed that the decreased viability and induced caspase 3/7 activity of IL-1β-induced OA-CH were reversed when Erk1/2, p38, and PI3K/AKT signaling were inhibited, confirming their role in IL-1β-induced catabolic effects.

In order to analyze the putative anti-inflammatory effects of Cur-EVs, their effect on critical pro-inflammatory signaling pathway components was investigated in IL-1β-stimulated OA-CH by determining the phosphorylation status of Erk1/2, PI3K/Akt, and p-38MAPK. Our data indicated that Cur-EVs could reduce IL-1β-induced phosphorylation of Erk1/2, PI3K/Akt, and p-38MAPK and thus reduce activity of pathways involving these kinases. These findings suggest that anti-inflammatory effects of Cur-EVs exist which might be transduced via modulation of IL-1β-induced pro-inflammatory signaling pathway activity.

Several studies confirmed that miRs are involved in the pathological processes of cartilage matrix degradation by regulating signaling pathways associated with inflammation [55–57]. We analyzed miR profiles from non-OA and OA cartilage biopsies and screened the most significant differentially expressed miRs with bioinformatic tools in public databases, leading to the identification of hsa-miR-126-3p as the most regulated miR in OA versus non-OA cartilage samples. In agreement with these data, when we compared the expression levels of hsa-miR-126-3p in non-OA-CH, OA-CH and IL-1β-induced OA-CH, the non-OA samples had the highest expression levels. Our data from transfection experiments with hsa-miR-126-3p mimic and inhibitors suggested that hsa-miR-126-3p reversed catabolic responses and reduced phosphorylation levels of Erk1/2, p38MAPK, and PI3K/AKT, which were induced by IL-1β treatment in OA-CH. From that, we assume that most likely, hsa-miR-126-3p is involved in modulating Erk1/2, p38MAPK, and PI3K/AKT-dependent signaling pathways. This is in line with data from KEGG enrichment of targeted genes identifying hsa-miR-126-3p to be involved in regulating angiogenesis, inflammation, and tumor growth [58–60]. Several studies demonstrated that hsa-miR-126-3p decreased cytokine release via inhibiting MAPK, NFκB, and PI3K/AKT signaling [58, 60–62]. It should be noted that there is literature indicating that miR-126 can also promote inflammatory responses in a chondrocyte cell line (CHON-001 cells) by activating NFκB and MAPK/JNK signaling pathways [63, 64]. This indicates that the underlying molecular mechanisms of hsa-miR-126-3p effects on pro-inflammatory signaling pathways in chondrocytes require further in-depth research, preferably in primary cells and in vivo rather than in cell lines.

According to the data of this study, the question arises do we suggest to rather apply miR-loaded EVs?

Several studies demonstrated that miRs could be efficiently loaded into the EVs [65–67]. Yujie et al. reported that EVs which are encapsulated with miR-140 alleviate OA progression via inhibiting ADAMTS-5 and MMP-13 [68]. Compared with Cur-EVs, the production process of miR loaded EVs (miR-EVs) requires additional steps to transfer nucleic acids to donor cells. Furthermore, in order to increase the loading efficiencies of miR-EVs, extensive chemical modifications will be needed to be performed, which requires expensive chemically modified oligonucleotides and reagents [69]. Accordingly, we would prefer to load EVs with curcumin and not with miRs as preparing Cur-EVs only requires to stimulate cells with curcumin.

In conclusion, our study demonstrated that Cur-EVs attenuated IL-1β-induced catabolic effects in OA-CH by promoting viability and migration and inhibiting apoptosis. In addition, expression of catabolic genes was downregulated whereas expression of anabolic genes was upregulated in IL-1β-induced OA-CH in the presence of Cur-EVs. The EVs prepared from curcumin-primed BMSC were clearly more effective than EVs from control BMSC and free curcumin, indicating that curcumin encapsulated in EVs potentiates the anabolic effects of BMSC-derived EVs. These effects may in part be attributed to upregulated expression of hsa-miR-126-3p in the target cells induced by Cur-EVs, with the consequence of reducing phosphorylation of components of pro-inflammatory signaling pathways. Our data indicated that EVs may be considered as a promising drug delivery vehicle of curcumin for treatment of OA, providing an efficient anti-inflammatory intervention strategy.

## Data Availability

The raw datasets (Datasets EMTAB-5715 and EMTAB-5716) used in this study were downloaded from ArrayExpress (https://www.ebi.ac.uk/arrayexpress/).

## Abbreviations

OA: Osteoarthritis
EVs: Extracellular vesicles
hBMSC: Human bone marrow-derived stroma cells
OA-CH: Osteoarthritic chondrocytes
CTB: Cell Titer Blue Viability Assay
Cur-EVs: Curcumin-primed extracellular vesicles
WB: Western blotting
IL-1β: Interleukin 1β
miR: MicroRNA
3’UTR: 3′-untranslated region
FCS: Fetal calf serum
NTA: Nanoparticle tracking analysis
TEM: Transmission electron microscopy
